# Predictive value of quantitative HER2, HER3 and p95HER2 levels in HER2-positive advanced breast cancer patients treated with lapatinib following progression on trastuzumab

**DOI:** 10.18632/oncotarget.22027

**Published:** 2017-10-24

**Authors:** Renata Duchnowska, Jeff Sperinde, Bogumiła Czartoryska-Arłukowicz, Paulina Myśliwiec, John Winslow, Barbara Radecka, Christos Petropoulos, Regina Demlova, Marlena Orlikowska, Anna Kowalczyk, Istvan Lang, Barbara Ziółkowska, Sylwia Dębska-Szmich, Monika Merdalska, Aleksandra Grela-Wojewoda, Anton Żawrocki, Wojciech Biernat, Weidong Huang, Jacek Jassem

**Affiliations:** ^1^ Military Institute of Medicine, Warsaw, Poland; ^2^ Monogram Biosciences, Integrated Oncology, Laboratory Corporation of America^®^ Holdings, South San Francisco, CA, USA; ^3^ Białystok Oncology Center, Białystok, Poland; ^4^ Oncology Center, Zielona Góra, Poland; ^5^ Opole Oncology Center, Opole, Poland; ^6^ Masaryk Memorial Cancer Institute, Brno, Czech Republic; ^7^ Warmia and Masuria Oncology Center, Olsztyn, Poland; ^8^ Medical University of Gdańsk, Gdańsk, Poland; ^9^ National Institute of Oncology, Budapest, Hungary; ^10^ Regional Hospital, Wrocław, Poland; ^11^ Medical University of Łódź, Łódź, Poland; ^12^ Oncology Center, Kielce, Poland; ^13^ Oncology Institute, Kraków, Poland

**Keywords:** breast cancer, trastuzumab, lapatinib, HER2, p95HER2

## Abstract

Lapatinib is a HER1 and HER2 tyrosine kinase inhibitor (TKI) approved in second line treatment of advanced or metastatic breast cancer following progression on trastuzumab-containing therapy. Biomarkers for activity of lapatinib and other TKIs are lacking.

Formalin-fixed, paraffin-embedded primary tumor samples were obtained from 189 HER2-positive patients treated with lapatinib plus capecitabine following progression on trastuzumab. The HERmark^®^ Breast Cancer Assay was used to quantify HER2 protein expression. HER3 and p95HER2 protein expression was quantified using the VeraTag^®^ technology.

Overall survival (OS) was inversely correlated with HER2 (HR = 1.9/log; P = 0.009) for patients with tumors above the cut-off positivity level by the HERmark assay. OS was significantly shorter for those with above median HER2 levels (HR = 1.7; P = 0.015) and trended shorter for those below the cut-off level of positivity by the HERmark assay (HR = 1.7; P = 0.057) compared to cases with moderate HER2 overexpression. The relationship between HER2 protein expression and OS was best captured with a U-shaped parabolic function (P = 0.004), with the best prognosis at moderate levels of HER2 protein overexpression. In a multivariate model including HER2, increasing p95HER2 expression was associated with longer OS (HR = 0.35/log; P = 0.027). Continuous HER3 did not significantly correlate with OS.

Patients with moderately overexpressed HER2 levels and high p95HER2 expression may have best outcomes while receiving lapatinib following progression on trastuzumab. Further study is warranted to explore the predictive utility of quantitative HER2 and p95HER2 in guiding HER2-directed therapies.

## INTRODUCTION

Lapatinib is a HER1 (human epidermal growth factor receptor type 1) and HER2 (type 2) tyrosine kinase inhibitor (TKI) first approved for treatment of advanced or metastatic breast cancer patients that had progressed on trastuzumab-containing therapy [1]. With the approval trastuzumab emtansine (TDM-1) and pertuzumab, lapatinib is less commonly used in second line treatment, but remains a valuable option in the third line setting and beyond.

Little is known about the factors that may cause sensitivity or resistance to lapatinib beyond HER2 status [2, 3]. Several biomarkers and clinical variables have been investigated, including serum HER2 extracellular domain [4, 5], p95HER2 truncated form of HER2 [6, 7] activation of the PI3K/AKT pathway [8], *EGFR* gene copy number [9], intrinsic breast cancer subtypes [10] and time to progression to first line trastuzumab [11], but none have found application in clinical practice. Discovery of additional biomarkers for lapatinib efficacy could potentially help guide treatment. Additionally, clinical development of other HER2 TKI’s, including neratinib (an irreversible tyrosine-kinase inhibitor of HER1, HER2, and HER4) [12] and tucatinib (a small molecule selective inhibitor of HER2) [13], may benefit from the discovery of predictive biomarkers.

In a previous study, we examined the association between lapatinib efficacy and downstream signaling markers, including phosphorylated adenosine monophosphate-activated protein (p-AMPK), phosphorylated mitogen-activated protein kinase (p-MAPK), phosphorylated p70 S6 kinase (p-p70S6K), hypoxia-inducible factor 2 alpha (HIF-2α), cyclin E, phosphatase and tensin homolog (PTEN), and estrogen receptor alpha (ERɑ) [14]. Elevated expression of p-MAPK and cyclin E correlated with shorter overall survival (OS), whereas ERɑ expression correlated with longer OS. In the current study, we sought to extend this work with the use of quantitative protein expression of HER2, HER3 and p95HER2, the hyperactive, truncated form of HER2.

## RESULTS

### Patient characteristics

Quantitative HER2, HER3 and p95HER2 protein expression was measured in FFPE samples obtained from the primary tumors of 189 patients treated with lapatinib plus capecitabine following progression on trastuzumab (Table 1). Patients received trastuzumab in the adjuvant (23%), metastatic (68%) or both settings (8%). Nearly all patients (98%) received chemotherapy. Thirteen percent of patients presented with metastatic disease at diagnosis. Twenty-six percent of patients had detectable brain metastases prior to receiving lapatinib.

**Table 1:** Patient characteristics

Characteristic	N	%
Median age at diagnosis, years (range)	56 (28-79)	
Hormone receptor status		
Negative	117	62
Positive	72	38
Grade		
1	4	2
2	64	34
3	72	38
Missing	49	26
Stage at diagnosis		
I	15	8
II	50	26
III	95	50
IV	25	13
Missing	4	2
Trastuzumab therapy		
Adjuvant	44	23
For advanced disease	129	68
Combination thereof	16	8
Brain metastases before lapatinib based therapy		
No	140	74
Yes	49	26

### Association of HER2, HER3 and p95HER2 with downstream signaling markers

**Figure 1:**
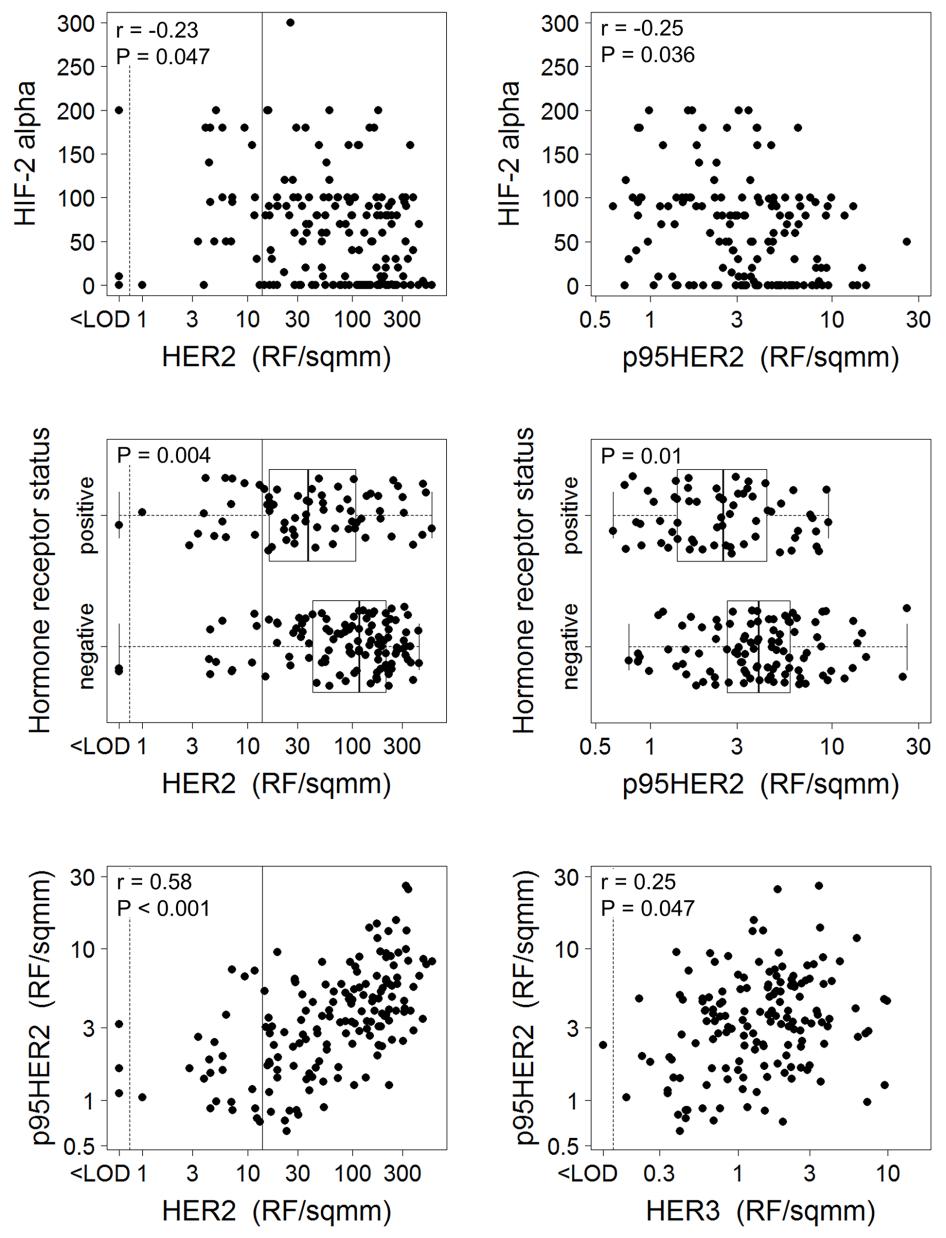
Significant relationships between biomarkers Spearman tests or a Mann-Whitney test in the case of hormone receptor status was used to examine correlations between biomarkers, corrected by Holm adjustment for multiple comparisons. Only the statistically significant relationships are shown. HER2 cut off shown for HER2 overexpression [27].

There was sufficient tumor to measure HER2, HER3 and p95HER2 in 182, 157 and 166 samples, respectively. A test for correlation was performed on all pairs of markers with adjustment of the p-value for multiple comparisons. The following markers were tested for pairwise correlations: HER2, HER3, p95HER2, p-AMPK, p-MAPK, p-p70S6K, HIF-2α, cyclin E, PTEN and hormone receptor status. Both HIF-2α and hormone receptor status were inversely correlated with HER2 and p95HER2, and p95HER2 was positively correlated with HER2 and HER3 (Figure 1). All other pairwise correlations were not statistically significant.

### Association of HER2, HER3 and p95HER2 expression with overall survival

**Figure 2:**
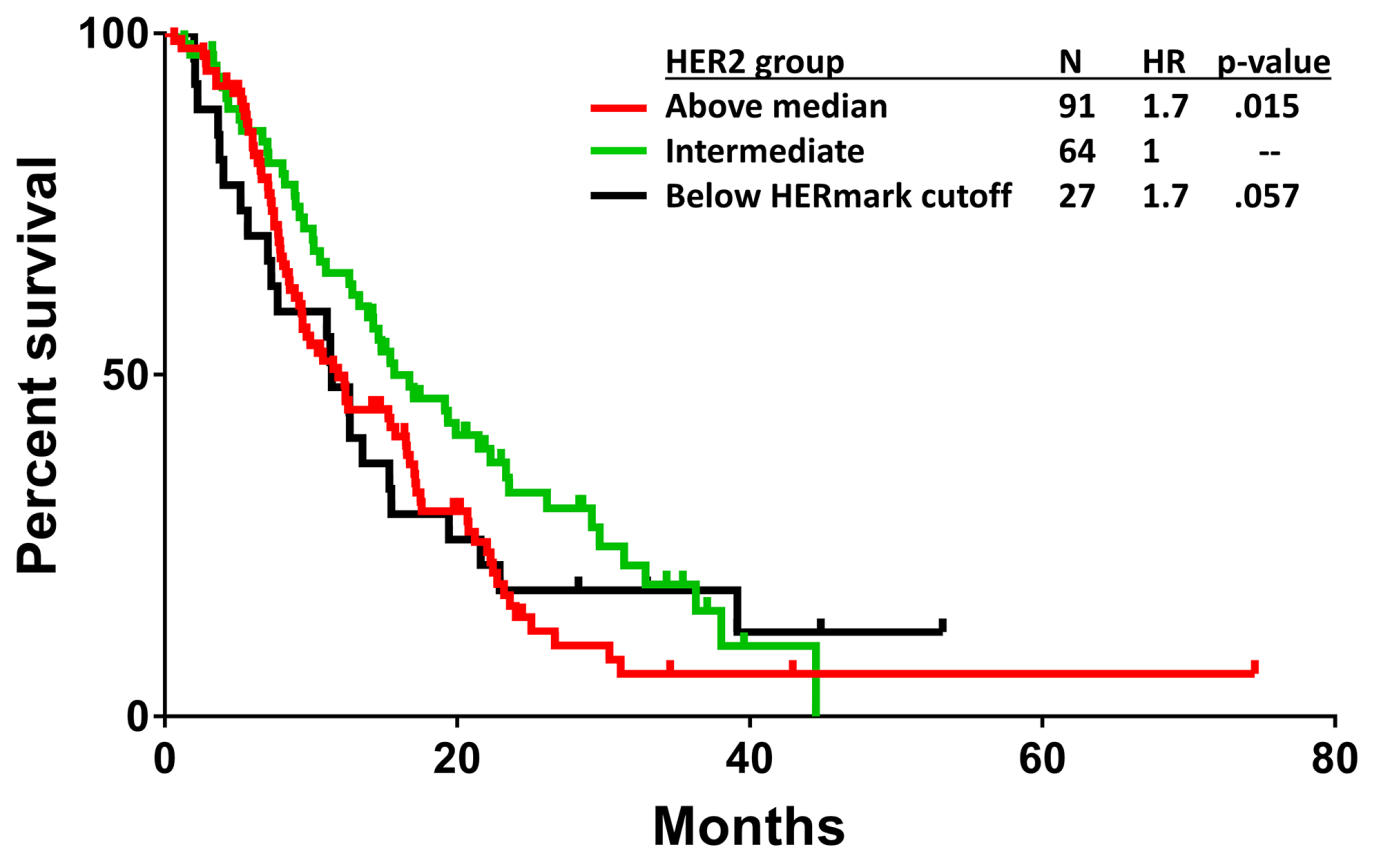
Kaplan-Meier OS plot by HER2 expression level Those with above-median HER2 (red) have a shorter OS than the intermediate group (green) with below-median HER2 but above the HERmark cutoff for overexpression (HR = 1.7; P = 0.015). Those that have normal HER2 expression by HERmark (black) trended toward shorter OS compared to the intermediate group in green (HR = 1.7; P = 0.057).

The HERmark HER2 assay is greater than 95% concordant with centrally determined HER2 status [15, 16]. In our study, 149 cases were positive by HERmark HER2 assay, 15 were equivocal and 22 were negative. We first examined the relationship between continuous HER2 and OS in the majority subset that overexpressed HER2 by HERmark. Increasing log(HER2) was found to correlate with shorter OS (HR = 1.9; P = 0.009). Further, those with above-median HER2 had a shorter OS than those with below-median HER2 (HR = 1.7; P = 0.015). We next considered the small subgroup that was discordant, i.e. had tumors with normal HER2 expression according to the HERmark assay, yet were HER2-positive by HER2 IHC or FISH. The group that had normal HER2 expression by HERmark trended toward shorter OS (HR = 1.7; P = 0.057), similar to the subgroup with above-median HER2 by HERmark (Figure 2). Consequently, the patients with moderately overexpressed HER2 had the longest OS compared to those with both above-median or normal HER2 expression by HERmark.

**Figure 3:**
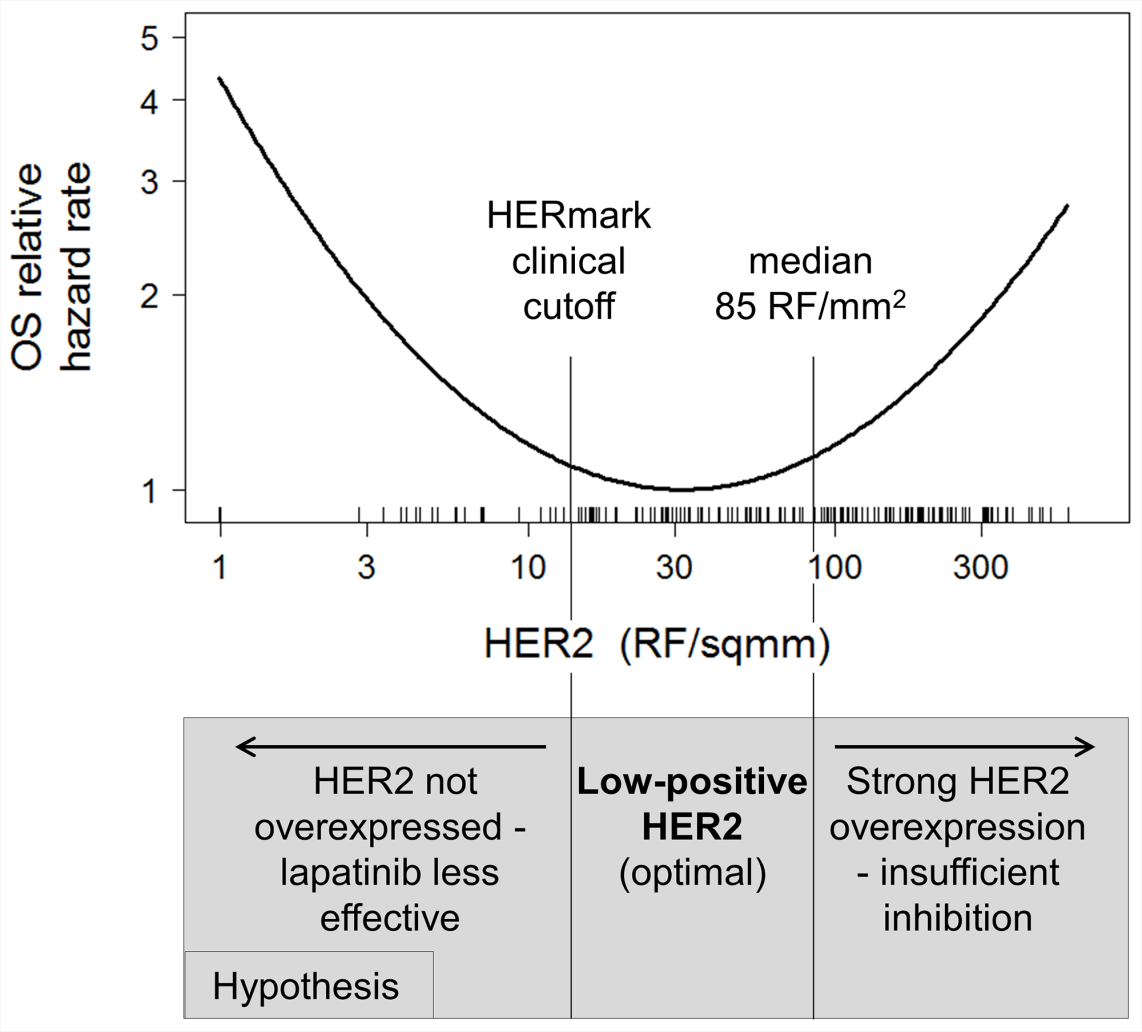
U-shaped relationship between HER2 and OS A parabolic equation, *Y = AX + BX*^*2*^, was tested in a Cox model where *Y* is the OS hazard rate, and *X* is log(HER2). This yielded significant coefficients *A* = -1.93 (P = 0.002) and *B* = 0.643 (P = 0.001), verifying that the parabolic function appropriately describes the relationship between HER2 and OS hazard rate.

This result implied a U-shaped or parabolic relationship between HER2 and OS. A simple parabolic equation can be written as *Y = AX + BX*^*2*^, where *Y* is the OS hazard rate, and *X* is log(HER2). This parabolic form of log(HER2) yielded statistically significant coefficients *A* = -1.93 (P = 0.002) and *B* = 0.643 (P = 0.001) in a Cox proportional hazards model, verifying the U-shaped relationship between HER2 and OS. Figure 3 shows a plot of the predicted OS hazard rate as a function of log(HER2) using the coefficients *A* and *B* derived from the Cox model. This model predicts an OS hazard rate minimum or OS optimum at a HER2 level of 32 RF/mm^2^, 2.3-fold above the HERmark cutoff for HER2 overexpression.

Continuous HER3 did not significantly correlate with OS (HR = 0.69; P = 0.18). However, in the hormone receptor negative subset and the subset of patients with above-median HER2 expression, increased HER3 trended with longer OS (HR = 0.52 and 0.48; P = 0.078 and 0.11, respectively).

**Figure 4:**
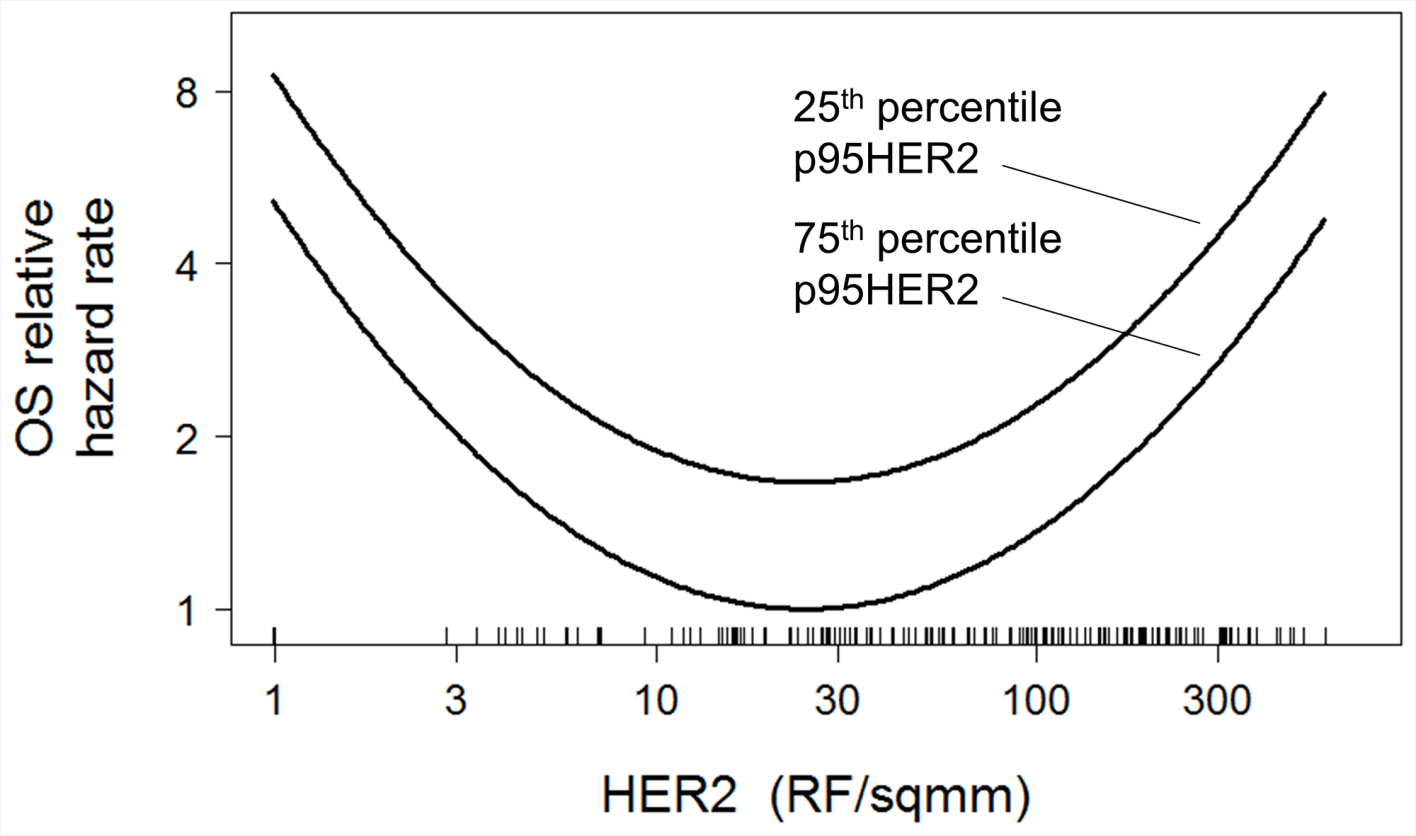
U-shaped relationship between HER2 and OS at either the 25^th^ or 75^th^ percentile of p95HER2 expression Parameters for curves were taken from Table 2 Cox model for patients with no pre-existing brain metastases.

p95HER2 was not correlated with OS both in the univariate (HR = 1.0; P = 0.98) and a multivariable model including HER2, p95HER2, HER3 and other clinical factors (HR = 0.62; P = 0.20) (Table 2). Presence of pre-existing brain metastases was found to be a strong predictor of shorter OS (HR = 2.4; P < 0.001). It was therefore hypothesized that excluding patients with pre-existing brain metastases might yield a clearer picture of influence of each biomarker on OS, since those patients universally progressed quickly. Indeed, in a multivariable model, increasing p95HER2 was found to correlate with longer OS (HR = 0.35; P = 0.027) in a subset of patients without pre-existing brain metastases (Table 2). Hormone receptor status and the U-shaped HER2 function (Figure 3) were also significantly correlated with OS in the multivariable model, while HER3 and stage were not. The combined effect of HER2 and p95HER2 expression levels on OS hazard rate in this subset can be interpreted as a U-shaped relationship between HER2 and OS, that shifts to lower hazard rates with increasing p95HER2 (Figure 4).

**Table 2:** Multivariable Cox models

	All patients (N = 146)		Patients with no pre-existing brain metastases (N = 103)	
Variable	HR	P-value	HR	P-value
Log(HER2)	--^*^	<0.001	--^*^	0.001
Log(HER2)^2^	--^*^	<0.001	--^*^	<0.001
Log(p95HER2)	0.62	0.20	0.35	0.027
Log(HER3)	0.80	0.42	0.86	0.65
Hormone receptor status	0.68	0.074	0.55	0.025
Stage	1.2	0.19	1.2	0.30
Pre-existing brain mets	2.4	<0.001	--	--

^*^HR varies with HER2. The shape of the relationship between HER2 and hazard rate is shown in Figure 3.

### Association of HER2, HER3 and p95HER2 expression with response

The association of quantitative HER2, HER3 and p95HER2 with RECIST response was examined using logistic regression. The median duration of lapatinib and capecitabine therapy was 6.2 months (range 0.2 - 52 months). The best response was complete response in 5% of patients, partial response in 33%, stable disease in 46% and progression in 15%. Only HER2 was significantly correlated with response (OR = 1.8; P = 0.033). There was no evidence for a better fit with a U-shaped function of HER2, in contrast to what was found for OS. The overall response rate was 13% in patients with normal HER2 expression by HERmark, 37% in those with below-median HER2 overexpression, and 45% in those with above-median HER2 overexpression.

## DISCUSSION

This study used quantitative measurements of HER2, HER3 and p95HER2 protein levels, thus allowing more in-depth analysis of their potential predictive relevance. A U-shaped curve best described the relationship between HER2 level and OS, with the longest OS occurring at a modestly overexpressed HER2 level (Figure 3). It is likely that different factors contributed to the shorter OS at each extreme of the HER2 spectrum. At the low end, there was a small number of cases with normal expression of HER2 by the HERmark assay, despite being HER2 positive by HER2 IHC or FISH, possibly due to tumor heterogeneity. These were a mixture of HER2 IHC 3+ with no FISH data and IHC 2+ with relatively low FISH/CEP17 ratio (median = 3.2). These cases with low HER2 expression by HERmark experienced shorter OS (Figure 2), possibly because HER2 was not the main driver of the tumor, making lapatinib less effective. Cases with HER2 overexpression above the median HERmark value also experienced shorter OS, possibly because lapatinib at the prescribed dose was not able to fully suppress HER2 activity.

The relationship between HER2 mRNA expression level and outcomes has been examined in other studies in this setting. In the EGF30008 trial, which examined letrozole with or without lapatinib in HER2-positive metastatic breast cancer, those with the highest HER2 mRNA levels did not benefit from lapatinib, as opposed to those with lower levels [17]. This mirrors the result of the current study for those that were HERmark-positive: moderate overexpression of HER2 was associated with the best prognosis, whereas high overexpression was associated with worse prognosis. In the EMILA trial, patients in the lapatinib plus capecitabine arm with below-median versus above-median HER2 mRNA expression were found to have similar overall response (30 vs. 31%), median PFS (6.4 vs. 6.9 months) and median OS (23.7 vs. 24.8 months) [18]. This is not inconsistent with a U-shaped relationship between HER2 and outcome if the median was near the inflection point, although the possibility of a U-shaped relationship was not discussed.

The response to lapatinib based on HER2 amplification level has also been examined. In a pooled analysis of EGF30001 and EFG100151, only HER2 amplified cases experienced longer PFS with the addition of lapatinib, and the degree of benefit did not vary with the degree of amplification [2]. Without HER2-directed therapy, increasing gene amplification correlates with shorter PFS [19]. Taken together, these findings imply that the survival in patients administered lapatinib may correlate with HER2 amplification level, with the best outcomes in those with a moderate amplification. Patients with no amplification have no benefit from lapatinib, whereas in those with high amplification the benefit from lapatinib may be masked by generally poor prognosis. This, apart from potentially insufficient HER2 suppression by lapatinib in this subset, aligns with the U-shaped relationship for OS observed in the current study.

In clinical practice, p95HER2 expression is not considered a criterion for anti-HER2 therapies, and is not measured [20]. Patients with high p95HER2 expression generally have high HER2 expression and receive trastuzumab as first line treatment, but may be less responsive since p95HER2 lacks the trastuzumab binding site. Lapatinib suppresses the activity of p95HER2 in cell lines and is equally active in breast cancer patients with tumors expressing low or high levels of p95HER2 [6, 7, 21]. Similarly, in the current study OS was independent of p95HER2 level in univariate analysis. However, in a multivariate analysis controlling for the influence of HER2 level, increasing p95HER2 expression correlated with longer OS (HR = 0.35; P = 0.027) in the subset without pre-existing brain metastases (Table 2 and Figure 4). Those with pre-existing brain metastases had a poor prognosis, regardless of p95HER2 level. The activity of lapatinib and capecitabine in brain metastasis is modest [22], though in radiotherapy-naïve patients it may delay the need of whole brain irradiation [23].

In our study, the correlation between HER2 and tumor response was different than that with OS. The contrast was particularly strong at the highest HER2 levels, where response was the highest, yet OS was the shortest. Hence, quantitative HER2 may be viewed in this setting as prognostic rather than predictive factor for lapatinib.

We have not demonstrated any significant relationship between continuous HER3 levels and lapatinib efficacy measured by OS or tumor response. HER3, as a co-receptor of HER2/HER3 heterodimers, plays a critical role in HER2-mediated transformation, tumor progression and resistance to HER TKIs, and its upregulation in experimental models was shown to limit inhibitory effect of these compounds [24]. Further, HER2 inhibition by lapatinib leads to a compensatory PI3K/Akt and FoxO3a-dependent upregulation of HER3 [25]. However, the relevance of these findings has not been investigated in a clinical setting.

Correlation of PFS with quantitative HER2, HER3 and p95HER2 expression has been examined in a similar cohort of trastuzumab-refractory patients treated with lapatinib and capecitabine [26]. No correlation with PFS was found for HER2 cutoffs, but a test for a potential U-shaped relationship with continuous HER2 was not performed. Similar to the current study, there was no correlation with HER3. A trend of longer PFS with increasing p95HER2 was noted especially in ER-positive cases, consistent with the significant correlation between increasing p95HER2 and longer OS in the current study.

In summary, the relationship between quantitative HER2 expression and OS for patients receiving lapatinib following progression on trastuzumab followed a U-shaped curve, with the best prognosis in those with moderate HER2 overexpression. P95HER2 was found to be an independent correlate of OS in a subset without pre-existing brain metastases. Our study included a homogenous group of patients treated with lapatinib and capecitabine, with relatively long follow-up. However, it also implies that a primary tumor biopsy was typically performed well ahead lapatinib administration. Additionally, a non-randomized study design does not allow for verifying genuine predictive value of particular markers. Hence, their prognostic and predictive relevance in HER2-directed therapies warrants further investigations.

## METHODS

### Patients

This study was approved by the Institutional Review Board of the coordinating center (the Military Institute of Medicine in Warsaw, Poland). The Study group included patients with HER2-positive advanced breast cancer treated with lapatinib plus capecitabine following progression on trastuzumab and 1-3 lines of chemotherapy.

### Assays

Quantitative HER3 expression was measured using a HER3 VeraTag assay [27], and HER2 expression was measured using the HERmark assay [15], which also uses the VeraTag method of proximity-based release of fluorescent tags. Briefly, HER2 or HER3 was quantified through the release of a fluorescent tag conjugated to a monoclonal antibody (mAb) via a linker that is sensitive to singlet oxygen. A second biotinylated mAb bound to an avidin-linked photosensitizer molecule produced singlet oxygen upon illumination with red light. Due to the short half-life of singlet oxygen, the tag was only cleaved when the two antibodies were bound in close proximity.

p95HER2 was determined using the p95HER2 VeraTag assay [28, 29]. Briefly, a mouse p95HER2 mAb specific for the active M611 carboxy-terminal fragment form of p95HER2 [30] was utilized with an anti-mouse secondary antibody conjugated to a fluorescent tag via a linker that is sensitive to reduction by dithiothreitol.

For the HERmark, HER3 and p95HER2 VeraTag assays, the released fluorescent tag was quantified by capillary electrophoresis and normalized to invasive tumor area, identified by a pathologist and quantified by image analysis software. The final units were relative fluorescence / mm^2^ of tumor (RF/mm^2^). Measurements were normalized to cell line standards of known expression levels. Cell lines used for normalization included MCF7, MDA-MB-453 and SK-BR-3 for HERmark, MDA-MB-231, MDA-MB-468 and MDA-MB-453 for HER3 and BT-474 plus two transfected cell lines for p95HER2. Each VeraTag assay batch was normalized by a weighted least squares fit to expected values for the three controls.

HER2 by HERmark, p95HER2 and HER3 levels were considered as continuous variables. Overexpression by HERmark HER2 was defined as >13.8 RF/mm^2^ [27] at the center of the HERmark analytical equivocal zone.

ER and progesterone (PR) expression was determined by IHC, with >10% nuclear staining considered positive. Tumors that were either ER or PR positive were considered hormone receptor positive.

### Statistical methods

Correlations between biomarkers were assessed by the Spearman rank correlation or Mann-Whitney U test in the case of hormone receptor status. P-values were adjusted for multiple comparisons by Holm’s method. Survival times were calculated from the initiation of lapatinib-containing therapy. Survival analyses were stratified by stage and presence of brain metastases prior to administration of lapatinib, except where these variables were used explicitly in multivariable analyses. Hazard ratios (HR) and odds ratios (OR) for continuous variables were given as the change in hazard per 10-fold change in the variable.
